# Ionization Radiation Shielding Effectiveness of Lead Acetate, Lead Nitrate, and Bismuth Nitrate-Doped Zinc Oxide Nanorods Thin Films: A Comparative Evaluation

**DOI:** 10.3390/ma15010003

**Published:** 2021-12-21

**Authors:** Mohamed Abdulsattar Al-Balushi, Naser M. Ahmed, Samer H. Zyoud, Mohammed Khalil Mohammed Ali, Hanan Akhdar, Osamah A. Aldaghri, Khalid Hassan Ibnaouf

**Affiliations:** 1 School of Physics, Universiti Sains Malaysia (USM), Gelugor 11800, Malaysia; mood.str@gmail.com (M.A.A.-B.); s.zyoud@ajman.ac.ae (S.H.Z.); 2 Department of Mathematics and Sciences, Ajman University, Ajman P.O. Box 346, United Arab Emirates; 3 Nonlinear Dynamics Research Center (NDRC), Ajman University, Ajman P.O. Box 346, United Arab Emirates; 4 Physics Department, College of Science, Imam Mohammad Ibn Saud Islamic University (IMSIU), Riyadh 13318, Saudi Arabia; hamofarog@yahoo.com (M.K.M.A.); odaghri@gmail.com (O.A.A.); kheo90@gmail.com (K.H.I.)

**Keywords:** ZnO-doping, radiation, X-ray, attenuation, chemical bath deposition

## Abstract

The fabrication of Nano-based shielding materials is an advancing research area in material sciences and nanotechnology. Although bulky lead-based products remain the primary choice for radiation protection, environmental disadvantages and high toxicity limit their potentials, necessitating less costly, compatible, eco-friendly, and light-weight alternatives. The theme of the presented investigation is to compare the ionization radiation shielding potentialities of the lead acetate (LA), lead nitrate (LN), and bismuth nitrate (BN)-doped zinc oxide nanorods-based thin films (ZONRs-TFs) produced via the chemical bath deposition (CBD) technique. The impact of the selected materials’ doping content on morphological and structural properties of ZONRs-TF was investigated. The X-ray diffractometer (XRD) analyses of both undoped and doped TFs revealed the existence of hexagonal quartzite crystal structures. The composition analysis by energy dispersive (EDX) detected the corrected elemental compositions of the deposited films. Field emission scanning electronic microscope (FESEM) images of the TFs showed highly porous and irregular surface morphologies of the randomly aligned NRs with cracks and voids. The undoped and 2 wt.% BN-doped TFs showed the smallest and largest grain size of 10.44 nm and 38.98 nm, respectively. The linear attenuation coefficient (µ) values of all the optimally doped ZONRs-TFs measured against the X-ray photon irradiation disclosed their excrement shielding potency. The measured µ values of the ZONRs-TFs displayed the trend of 1 wt.% LA-doped TF > 1 wt.% LN-doped TF > 3 wt.% BN-doped TF > undoped TFs). The values of μ of the ZONRs-TFs can be customized by adjusting the doping contents, which in turn controls the thickness and morphology of the TFs. In short, the proposed new types of the LA-, LN- and BN-doped ZONRs-TFs may contribute towards the development of the prospective ionization radiation shielding materials.

## 1. Introduction

Various ionizing radiations from different sources are responsible for the elevated risk of multiple types of cancers in humans worldwide [1,2,3]. Thus, radiation safety is one of the major concerns in medical imaging and industry. To avoid hazardous X-rays, radiographers and patients commonly use protective aprons made from lead (Pb) to protect themselves from harmful radiation [4]. The high density of Pb makes it a potential shielding material to efficiently absorb and attenuate the traversing X-ray photons [5,6,7]. However, the toxicity and heavy nature of the metal Pb remain significant limitations. Over the years, constant research efforts were made to reduce Pb usage and find alternative nontoxic and lighter materials. The European Union banned Pb usage in the healthcare sector, thereby creating the urgent necessity of a Pb substitute candidate [8]. Alternatively, various composite materials were proposed as effective absorbers and attenuators for the X-ray and Gamma-ray irradiation [9,10]. These shielding materials must have the capacity to attenuate the ionizing radiations passing through them thoroughly, thereby minimizing the risk of exposure to workers and people around. Nowadays, various nanoparticles-based composite materials are combined with a low fraction of Pb to achieve a light-weight X-ray shield. In this regard, nanofilms of various materials became prospective in the X-ray shielding aprons owing to their reduced toxicity, inexpensiveness, light-weight, and low production cost [11,12]. Different types of nanomaterials were utilized to develop radiation shielding with enhanced performance compared to ordinary materials. For instance, Ambika et al. reported the attenuation coefficient (µ) for isophthalic resin filled with different percentages of bismuth oxide (Bi_2_O_3_), the source of radiation used was gamma photons with 662 keV energy emitted from Caesium-137 (Cs-137) [13]. The results demonstrated an increment of attenuation coefficient (µ) with increasing the Bi_2_O_3_; also, the attenuation coefficient (µ) increased with the increasing of the density of filler polymer composite. Later, M. Vagheian et al. employed Monte–Carlo computational and experimental methods to investigate the X-ray shielding properties of bulk and nanostructured thin lead films. In this case, lead samples of different thicknesses; 10, 100, and 1000 nm were fabricated using Physical Vapor Deposition (PVD) technique. Subsequently, the prepared samples were exposed to X-Ray energy in the range of 8–14 KeV [14]. Further, the shielding properties of the bulk-structured thin films was evaluated using Monte-Carlo MCNPX code. At low energies, the results demonstrated better attenuation potential for nanostructured lead thin films compared to that of bulk-structured samples. But the variance vanishes when the film thickness was increased to 1000 nm. Similar behavior was observed at the X-ray energy of 14 keV.

**Figure 1:**
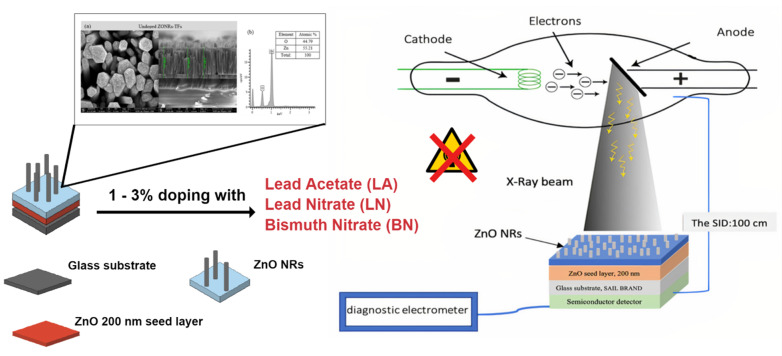
A graphical representation summarizes central idea of this work.

Zinc oxide (ZnO) is one of the highly utilized materials for industrial and biomedical applications. The ease of preparation and the elasticity in various morphologies with properties made such material very appealing [15]. Considering the immense fundamental and increasing interests of the doped ZnO nanofilms. This work is aimed at reporting a comparative study between three different ZnO-based materials and investigation of their X-ray radiation shielding properties. We successfully synthesized and characterized three series of the ZnO nanorods thin films (ZONRs-TFs) doped at various contents (1, 2, and 3 wt.%) with lead acetate (LA), lead nitrate (LN), and bismuth nitrate (BN) using chemical bath deposition (CBD) (Figure 1) Due to the CBD’s several advantages, it was chosen to grow the proposed thin films (TFs) on the glass substrates [16,17]. The findings showed the feasibility of customizing the ionization radiation shielding potential of the proposed Zinc oxide Nano-rods thin films (ZONRs-TFs) by tuning the doping contents of LA, LN, and BN. The proposed new types of the LA-, LN-, and BN-doped ZONRs-TFs may contribute to developing the prospective ionization radiation shielding materials.

## 2. Materials and Methods

### 2.1. Materials

High purity chemical reagents of zinc nitrate tetrahydrate salt [Zn(NO_3_)_2_·4H_2_O] with the MW of 261.44 g mol^−1^ (Merck, Darmstadt, Germany), hexamine hexamethylenetetramine salt [C_6_H_12_N_4_] with the MW of 140.19 g mol^−1^ (Scharlau, Cham, Germany), and deionized water were used to prepare the ZnO solution. The LA salt [Pb (CH3COO)_2_·3H_2_O] with the MW of 379.33 g mol^−1^ (HmbG, Hamburg, Germany), LN salt [Pb(NO_3_)_2_] with MW of 331.21 g mol^−1^ (Avonchem, Cheshire, UK), and BN salt [Bi(NO_3_)_3_·5H_2_O] with MW of 485.07 g mol^−1^ (QRec, Rawang, Malaysia). All the mentioned materials were used without any purification.

### 2.2. Synthesis of ZnONR-TFs Using CBD Technique

**Figure 2:**
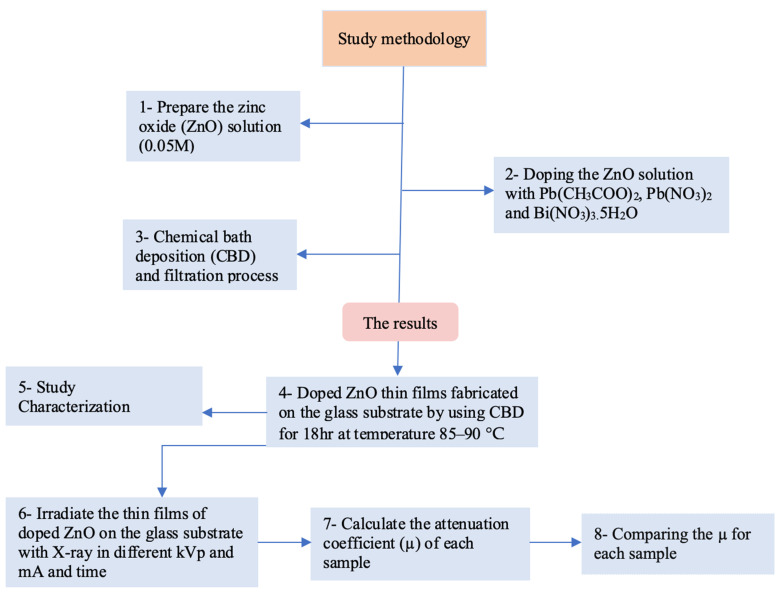
Flowchart of study methodology.

**Figure 3:**
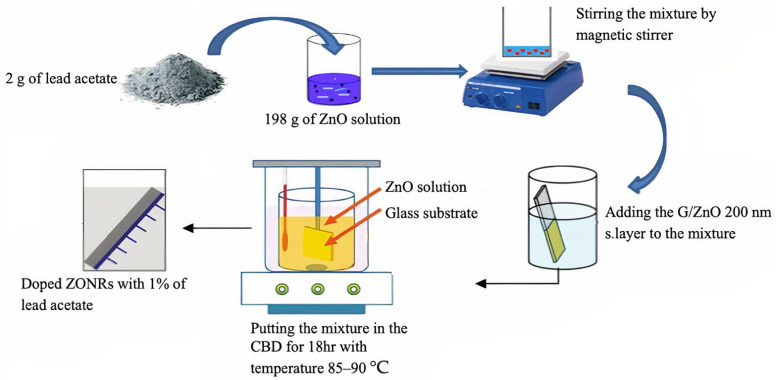
Various stages of sample preparation via CBD.

Figure 2 depicts the methodology followed for the present work. ZONRs-TFs were synthesized on the glass substrate using the CBD technique (Figure S1). The samples were prepared in four stages (Figure 3) Firstly, the ZnO seed layer of thickness nearly 200 nm was deposited on the glass substrate via the radiofrequency reactive sputtering (RFRS). Secondly, the ZnO solution was made from Zinc nitrate tetrahydrate (Zn(NO_3_)_2_·4H_2_O) ZNTH and hexamethylene-tetraamine HMTA by dissolving an equimolar (0.05 M) HMTA to ZNHT in 0.2 L of Deionized water (DIW), followed by continuous stirring for 1 h at room temperature achieve a homogenous mixture. Thirdly, the varying levels (1, 2, and 3 wt.%) of LA, LN, and BN were added separately to the prepared ZnO solution to obtain the ZONRs-TFs via CBD. In this process, the LA of 2 g (1 wt.%) was doped in 198 g of ZnO followed by mixing the magnetic stirrer for 2 h at room temperature to get the homogeneous mixture. Finally, the glass substrate was coated with the ZnO seed layer of 200 nm thick was placed within the resultant solution before being transferred to the CBD unit. The deposition was conducted in the temperature range of 85–90 °C for 18 h to get the ZONRs-TFs (Figure S2). An identical procedure was followed at each doping level to get nine samples with three in each series, as shown in Table 1.

### 2.3. Characterization of as Made ZnONR-TFs

X-ray diffractometer (XRD) (Bruker D8 Advance, AXS GmbH, Karlsruhe, Germany) was utilized to study the structure of prepared undoped and doped ZONRs-TFs films, with the X-ray sources of (Cu K1) line of wavelength 1.54 Å being used. The field emission scanning electron microscope (FESEM, FEI Nova SEM 450, FEI Company, Hillsboro, OR, USA) was used to image the morphology of the samples. The trace elements in the samples were detected using the energy-dispersive X-ray (EDX) spectrometer. To irradiate samples with X-ray photon beam, 50–100 kVp operated from Toshiba X-ray KXO-50S, the general radiography unit available in the medical physics lab, Universiti Sains Malaysia, was used.

## 3. Results and Discussion

### 3.1. Morphological and Structural Characterization of ZONRs-TFs

**Figure 4:**
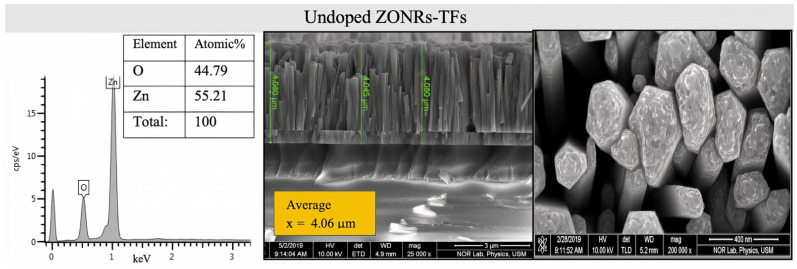
Cross-sectional FESEM images (**right**) and EDX spectra (**left**) of undoped ZONRs-TFs.

**Figure 5:**
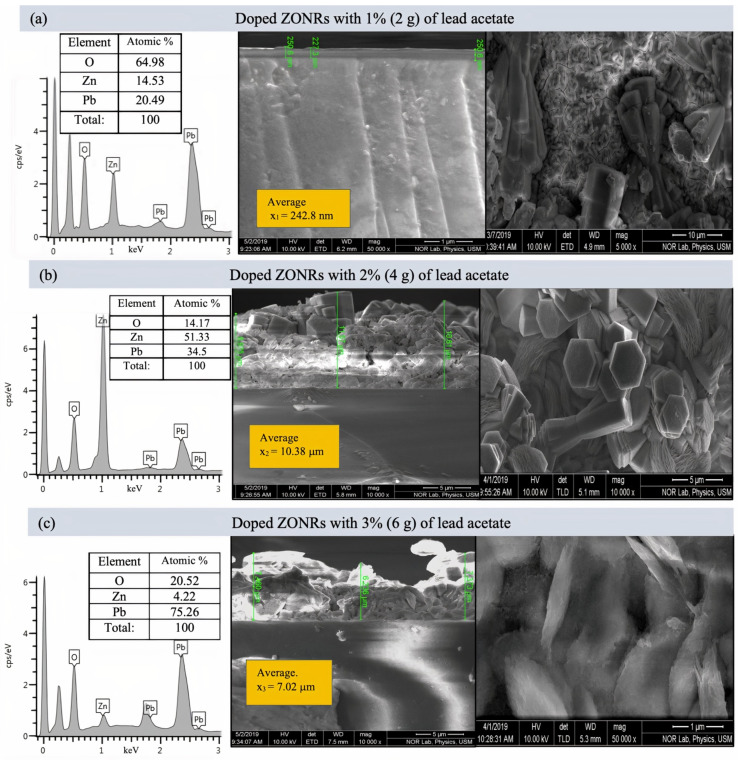
Cross-sectional FESEM images (**right**) and EDX spectra (**left**) of the ZONRs-TFs doped with 1–3 wt.% of LA, Inset: EDX Atomic% of detected trace elements.

**Figure 6:**
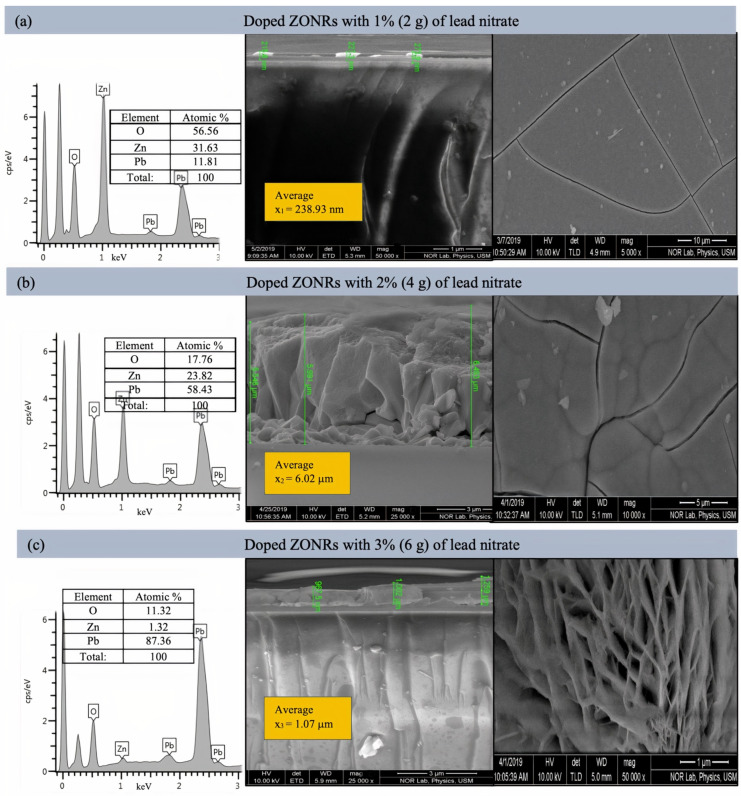
Cross-sectional FESEM images (**right**) and EDX spectra (**left**) of ZONRs-TFs doped with 1–3 wt.% of LN. Inset: EDX Atomic% of detected trace elements.

**Figure 7:**
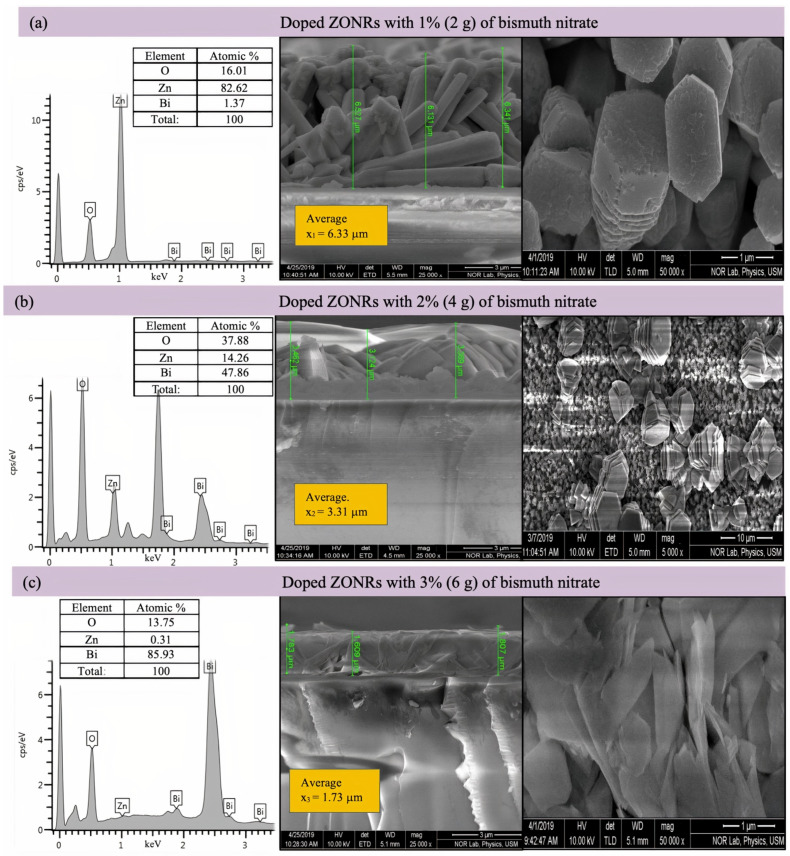
Cross-sectional FESEM images (**right**) and EDX spectra (**left**) of ZONRs-TFs doped with 1–3 wt.% of BN. Inset: EDX Atomic% of detected trace elements.

Figure 4 illustrates the cross-sectional FESEM micrograph and the EDX spectra of the undoped ZONRs-TFs. The Field emission scanning electron microscopy (FESEM) morphology consisted of dense and aligned hexagonal ZONRs with near-uniform distribution on the substrate surface. The inset of Figure 4 displays the nucleation of ZONRs in the TF with an average thickness of 4.068 μm. The EDX spectra detected the sample’s major trace elements (Zn and O) (inset Table in Figure 4). The EDX spectra and FESEM images of the ZONRs-TFs prepared with different contents of LA doping are shown in Figure 5a–c, LN doping in Figure 6a–c, and BN doping in Figure 7a–c. The doping contents had significant effects on the structures and morphologies of the samples. Therefore, sampling containing 1 wt.% was restricted to a small number of elements (Pb and Bi) to avoid aggregation and ensure uniform thin film distribution on the glass substrate.

Samples containing 2 and 3 wt.% of the LA and LN doping were agglomerates and highly porous with random orientation of the Nano-Rods (NRs) than the one made with 1 wt.% of LA. While samples containing 1 and 2 wt.% of the BN doping showed higher porosity, voids, and randomly oriented NRs than those synthesized with 3 wt.% of BN (revealed lower porosity and sheets-like morphologies). This indicated that it was easier for the X-ray photons to traverse through the TFs with higher porosity due to lower attenuation or weak absorption than those with lower porosity. Thus, the ZONRs-TFs prepared with 1 wt.% of the LA and LN could be the optimum shielding material than the presence of appropriate chemical elements (O, Zn, and Pb) in all the samples, while 3 wt.% of the BN doping was chosen as the optimum sample for the X-radiation shielding evaluation. The Atomic% of the O, Zn, and Pb in the ZONRs-TFs (inset of the EDX spectra) prepared with 1 wt.% of the LA, LN, and BN was also displayed in Figure 6 and Figure 7. These values were altered for the samples obtained with 2 wt.% and 3 wt.% of LA, indicating the role of LA contents on the morphologies and structures of the ZONRs-TFs. Besides, the amount of Pb was increased with the LA doping levels, improving the proposed material’s shielding potential. The higher amount of Pb in the TFs could enhance their X-ray photons attenuation capacity due to the higher atomic number and density of the element compared to the other constituents in the different elements present in the TFs. Table 2 displays the LA, LN, and BN doping concentration-dependent average thickness of the ZONRs-TFs obtained from the cross-section done by FESEM image analysis. In this study, three thickness values were measured, and then the average was calculated for each sample as shown in the (Figure 5, Figure 6 and Figure 7). Finally, the standard deviation was calculated for each sample by using Equation (1):

(1) $$\sigma =\sqrt{\frac{\sum {\left(x-\bar{x}\right)}^{2}}{n-1}}$$

where *σ* donates the standard deviation, *n* is the number of samples or thickness, *x* is the individual thickness values, and the $\bar{x}$ is the mean or the average value due to the non-uniformity or unevenness with cracks and voids of the FESEM surface morphologies. The observed thickness fluctuation due to the nonuniform distributions of the NRs on the glass substrate was attributed to the LA concentration-dependent structural and morphological alterations that occurred during the deposition at a high bath temperature for an extended period [18,19,20]. The average thickness of the ZONRs-TFs obtained with 2 and 3 wt.% of BN was much lower than those achieved with LA and LN doping.

**Figure 8:**
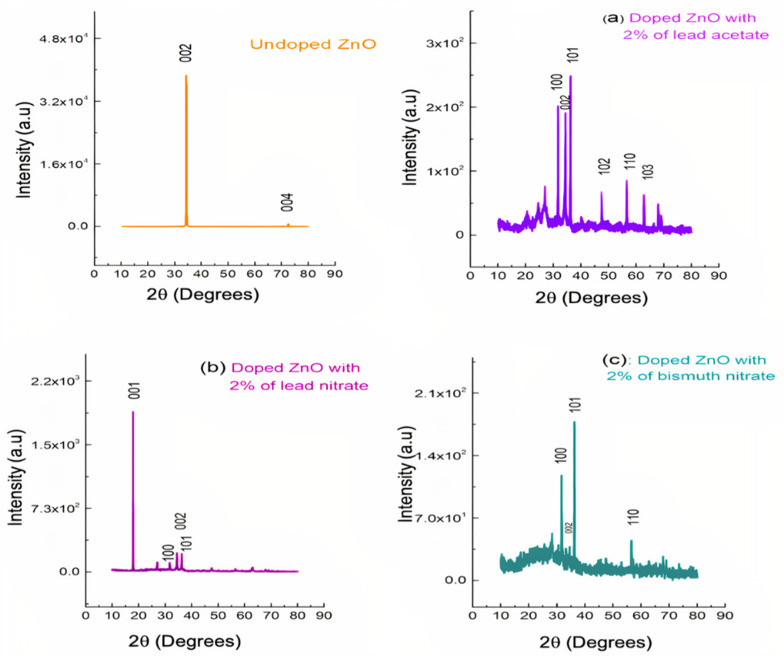
XRD patterns of undoped and doped ZONRs-TFs made with 2 wt.% of (**a**) LA, (**b**), LN, and (**c**) BN.

The XRD patterns of the as-prepared undoped and doped ZONRs-TFs are shown in Figure 8 The undoped sample’s intense XRD peak indicated the preferred lattice growth orientation along the (002) and (004) crystalline planes. However, the LA-, LN-, and BN-doped samples revealed several sharp peaks, indicating the preferred lattice growth orientations along the (001), (100), (101), (002), (102), (110), and (103) crystalline planes. The achieved high crystallinities of the TFs were suitable for the X-radiation shielding. The diffraction peaks corresponding to the (001), (100), (101), and (002) lattice orientations were the most intense, indicating their preferential crystal growth along these planes. For the undoped sample, the crystalline diffraction peaks corresponding to the growth orientations of (002) and (004) planes occurred at an angle (2θ) of 34.364 and 72.431°, respectively. However, the (002) peak decreased, and the (004) peak disappeared due to doping. The ZONRs-TFs prepared with 2 wt.% of LA showed more peaks (better crystallinity) than those doped with 2 wt.% of LN and BN. In short, the crystallinity and the lattice structures were appreciably affected by the nature of dopants due to the atomic masses and radii of the dopants. The crystallite diameter (grain size) in each sample corresponding to the intense XRD peak (preferred growth planes) was calculated using Scherrer’s equation given by:

(2) $$D=\frac{0.89\lambda }{\beta \;\cos\theta }$$

where *D* denotes the average crystallite size, *λ* is the X-ray wavelength (0.15406 nm), θ signifies the Bragg’s angle corresponding to the (002) growth direction, and β indicates the full width at half maximum (FWHM) of the intense diffraction peak.

Table 3 enlists the estimated size of the grains present. Overall, the grain size was increased with increases in dopants from lead-based to bismuth-based, which may be due to the difference in their atomic mass and radii.

### 3.2. Evaluating the Radiation Attenuation Capability of ZONRs-TFs

**Figure 9:**
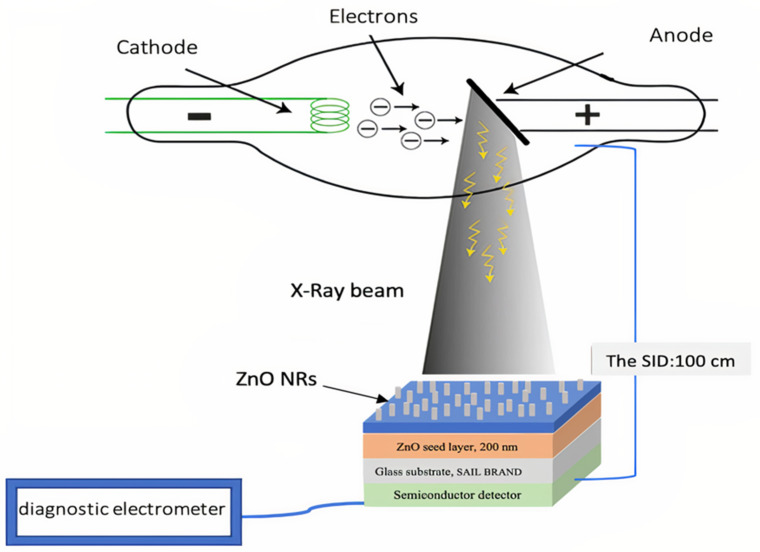
Schematic diagram of Experimental Setup.

Low-energy X-rays were used to irradiate the optimum ZONRs-TFs Incident ($I_{o}$) and transmitted (I) intensities of the photons were recorded without and with the thickness sample (x) during the study respectively via the diagnostic detector linked to the semiconductor dosimeter [20,21]. The X-ray tube operated with the respective voltage in the range of 50–100 kV (Tables S1–S3), and the current range of 50–200 mA was separated from the Source to image distance (SID) detector by 100 cm, as shown in Figure 9.

The values of attenuation (µ) were estimated via the relations [21]:

(3) $$I=I_{o}e^{-x\mu }$$

(4) $$\mu =\frac{\ln\frac{I_{o}}{I}}{x}$$

**Figure 10:**
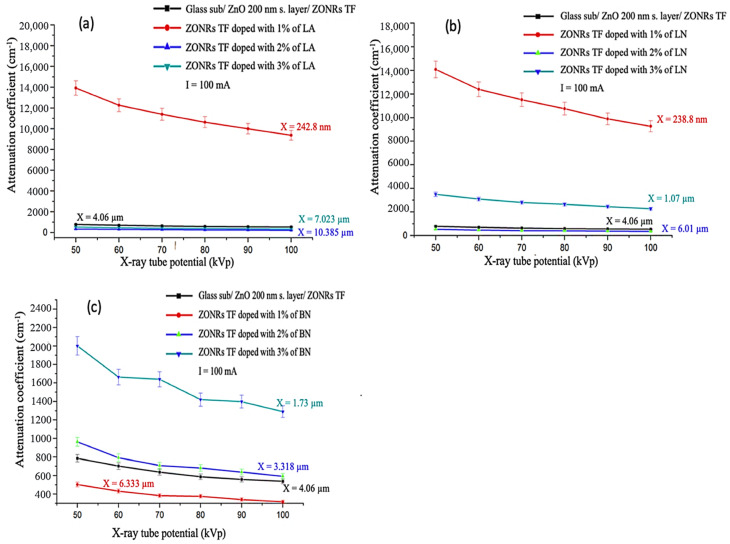
Linear attenuation coefficient (µ) vs. X-ray tube voltages for undoped ZONRs and doped with 1%, 2%, and 3% of LA (**a**), LN (**b**), and BN (**c**), current of tube is 100 mA.

Figure 10a–c illustrates the obvious difference in X-ray attenuation (*µ*) between undoped ZONRs-TFs and doped ZONRs-TFs with 1%, 2%, and 3% of LA, LN, and BN, respectively. The X-ray beams were generated in the range of (50–100) kV tube voltage and current 100 mA, (more details about the Effect of the Tube Current (mA) on the linear attenuation coefficient (*µ*) in Figures S3–S5). The result shows that the thin films (Nano and microscale) with less thickness have higher *μ* than each material. For instance, Figure 10a shows the *μ* of doped ZONR-TF with 1% of LA (thickness x = 242.8 nm) higher than *μ* of undoped ZONRs-TF (x = 4.06 µm). Moreover, it is also higher than the *μ* of doped ZONRs-TF with 3% of LA (x = 7.02 µm) and higher than the *μ* of doped ZONRs-TF with 2% of LA (x = 10.385 µm) [22].

**Figure 11:**
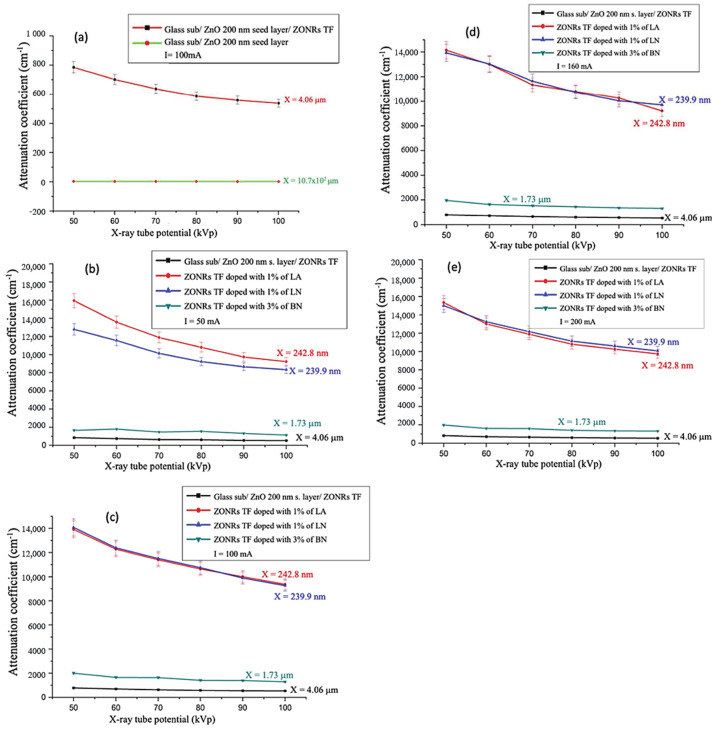
Irradiation energy dependent variation of μ for 200 nm-thick ZnO seed layer (**a**) undoped ZONRs-TFs obtained at tube current of 100 mA, (**b**–**e**) optimally doped ZONRs-TFs obtained at current (50 mA, 100 mA, 160 mA, and 200 mA).

The X-ray irradiation energy-dependent variation of μ for the 200 nm (x = 4.06 μm) thick ZnO seed layer and undoped ZONRs-TFs were obtained, shown in Figure 11a. The achieved values of μ for both seed layer and undoped ZONRs-TFs were high, indicating their strong radiation shielding potential. Present findings are in good agreement with the earlier observation on the ZnO-based novel composites for the γ-ray shielding applications [23,24]. For the undoped ZONRs-TFs and the optimum 1 wt.% LA-, 1 wt.% LN-, and 3 wt.% BN-doped ZONRs-TFs at the tube current of 50, 100, 160, and 200 mA, respectively, as shown in Figure 11b–e. Generally, at lower tube current, the results of the attenuation coefficient for the proposed ZONRs-TFs revealed the trend of μ values of 1 wt.% LA-doped TF > 1 wt.% LN-doped TF > 3 wt.% BN-doped TF > undoped TF. However, at higher tube current, the μ values of the 1 wt.% LA-doped ZONRs-TF and 1 wt.% LN-doped ZONRs-TF showed a slight crossover which may be due to different crystallinity, thickness, and morphologies of these two samples. Furthermore, the values μ for all the TFs were decreased with the X-ray tube voltages. This implied that the X-ray photons with higher energy have a greater chance of penetrating through the proposed ZONRs-TFs than those with lower energy [25,26].

### 3.3. Effect of Film Thickness and Porosity on µ of Optimally Doped ZONRs-TFs

Table 4 Depicts the measured values of µ (cm^−1^) for the optimum LA-, LN- and BN-doped ZONRs-TFs (with different thicknesses) at different X-ray photon energies (tube voltages and currents). The optimum ZONRs-TF prepared with 1 wt.% of LA revealed the highest value of µ in the studied voltage range at a low current of 50 mA. At the other tube currents, the optimum ZONRs-TF containing 1 wt.% of LN revealed the highest value of µ in the voltage range, indicating the effects of the film thickness on the attenuation or absorption of the X-ray photons. This result was consistent with the previously reported study related to the X-ray photons’ transmission at lower radiation energies through the nanosized and micro-sized TFs [20]. It was demonstrated that the micro-sized TFs had higher porosity and lower density than the nanosized TFs. The porosity of the shielding material was argued to be a significant factor for X-ray radiation absorption and attenuation. It was also acknowledged that the nanofibrous materials’ porosity has remarkable effects on the X-ray photons’ attenuation traits. The higher porosity caused lower attenuation of the photons when passed through the absorber than the one with lower porosity. Moreover, with a decrease in wt.%, the filler size will decrease. The packing density increases as the Nanofillers occupy all the interstitial spaces, unlike micron-sized, which create voids in the matrix. [27] Also, the interparticle distances for nanoparticles are much lower than the microparticles. Consequently, the void paths which allow photons to penetrate are reduced, which results in higher photon attenuation [28]. Nevertheless, the nanostructured materials (especially the nanoparticles, nanorods, nanofibers, and nanofilms) with more regular and smaller agglomeration are favorable for the attenuation enhancement high-quality shielding candidate [29,30]. Additionally, the higher surface to volume ratio for nanoparticles compared to microparticles increases the probability of interactions between the radiation and nanoparticles. This is in addition to greater homogeneity of composites incorporating nanoparticles compared to micro particles [31].

### 3.4. Comparative Insights to the Findings

For the sake of performance comparison, several studies were reported seeking the similar aim, for instance, N. N. Azman et al., 2013 examined the effect of tube voltage on the X-ray transmission in tungsten-epoxy composite fabricated by melt mixing sample using micro and nanosized structures. A low and high energy X-ray photon produced by mammography and general radiography unit were used. It was reported that with lower tube voltage (25–35 kV) and the nanosized tungsten oxide better attenuation was achieved with respect to microsized tungsten oxide. Meanwhile, using higher tube voltage (40–120 kV), the effect of particle size of tungsten oxide (WO3) was negligible [31] However, in our study, the nano and micro thin films were irradiated using tube voltage (50–100 kVp) revealed decent X-ray attenuation outcomes for optimum samples. Furthermore, M. Vagheian et al. investigated the X-ray shielding properties of bulk and nanostructured thin lead films by means of Monte-Carlo computational and experimental methods, respectively. The findings indicated that for low X-ray energies, the nanostructured lead thin films attenuate more than the bulk-structured samples; however, the difference disappears as film thickness increases to 1000 nm or X-ray energy reaches 14 keV. At variance, the optimized samples fabricated in this work demonstrated the capability to attenuate X-ray more than samples mentioned in the previous studies [14].

## 4. Conclusions

In summary, three series of LA-, LN-, and BN-doped (varying wt.%) ZONRs-TFs together with the undoped ZONRs-TFs were grown on the glass substrates via the conventional CBD and characterized using different techniques. The structures and morphologies of the prepared nanocomposite films were evaluated to determine the feasibility of getting the best X-ray photons absorber as a novel shielding material. Besides, the X-ray photons shielding potency of the LA-, LN-, and BN-doped optimum ZONRs-TFs were compared. The dependence of the attenuation coefficient of the optimum samples on the TF thickness, X-ray tube voltages (50–100 kVp), and currents (50 to 200 mA) was determined. The doping contents variation significantly affected the structures, morphologies, thickness, and ionization radiation shielding properties of the proposed ZONRs-TFs. XRD analyses of both the undoped and doped TFs revealed the existence of hexagonal quartzite crystal structures. The EDX analyses detected the corrected elemental compositions of the deposited films. The FESEM images of the TFs showed highly porous and irregular surface morphologies of the randomly aligned NRs with cracks and voids. The nanosized TFs (lower porosity and better absorber) exhibited higher values of μ than the micron-sized TFs (higher porosity and poor absorber). It is asserted that the values of μ of the ZONRs-TFs can be customized by adjusting the doping contents, which in turn controls the thickness and morphology of the TFs. In short, the proposed new types of the LA-, LN- and BN-doped ZONRs-TFs may contribute to developing the prospective ionization radiation shielding materials. In short, we envision this study contributing to the development of prospective ionization radiation shielding materials.

## Figures and Tables

**Table 1:** Weight% of doping agents (LA, LN, and BN) mixed in ZnO solution.

Doping (wt.%)	Mass of Each Dopant (g)	Mass of ZnO (g)	Total Mass in Solution (g)
1	2	198	200
2	4	196	200
3	6	194	200

**Table 2:** Average thickness of LA-, LN-, and BN-doped ZONRs-TFs was obtained from FESEM image analysis.

No	Sample	Thickness			Average	Standard Deviation	Thickness in (cm)
		X_1_	X_2_	X_3_			
**LA-doped ZONRs-TFs**							
1	Doped 1%	250.6	227.3	250.5	242.8 nm	±13.4 nm	2.42 × 10^−5^
2	Doped 2%	8.975	11.57	10.61	10.38 µm	±1.31 µm	10.38 × 10^−4^
3	Doped 3%	7.46	6.23	7.37	7.02 µm	±0.68 µm	7.02 × 10^−4^
**LN-doped ZONRs-TFs**							
1	Doped 1%	215.6	227.3	273.9	238.93 nm	±30.84 nm	2.39 × 10^−5^
2	Doped 2%	5.548	5.991	6.493	6.01 µm	±0.47 µm	6.01 × 10^−4^
3	Doped 3%	0.9675	1.002	1.259	1.07 µm	±0.15 µm	1.07 × 10^−4^
**BN-doped ZONRs-TFs**							
1	Doped 1%	6.527	6.131	6.341	6.333 µm	±0.198 µm	6.33 × 10^−4^
2	Doped 2%	3.462	3.124	3.369	3.318 µm	±0.174 µm	3.31 × 10^−4^
3	Doped 3%	1.783	1.609	1.807	1.730 µm	±0.108 µm	1.73 × 10^−4^

**Table 3:** Grain sizes of undoped and LA-, LN-, and BN-doped (2 wt.%) ZONRs-TFs.

ZONRs-TFs	Undoped	Doped of LA	Doped of LN	Doped of BN
Grain size (nm)	10.44	13.86	13.72	38.98

**Table 4:** Measured values of μ (cm^−1^) for optimum LA-, LN-, and BN-doped ZONRs-TFs at different X-ray photon energies and tube currents.

Doped ZONR-TFs with	Thickness	I (mA)	μ (cm^−1^) at Tube Voltage		
			50 kVp	70 kVp	100 kVp
1 wt.% of LA	242.8 nm	50	15,936.47	11,887.69	9220.81
		100	13,920.34	11,394.77	9376.06
		160	14,152.49	11,340.37	9220.81
		200	15,333.21	11,887.69	9740.59
1 wt.% of LN	239.9 nm	50	12,777.47	10,138.64	8331.74
		100	14,078.34	11,510.6	9264.97
		160	13,939.1	11,643.5	9702.78
		200	15,000.34	12,167.46	10,064.86
3 wt.% of BN	1.73 μm	50	1662.9	1473.37	1122.29
		100	2001.6	1639.16	1289.85
		160	1962.47	1530.01	1308.98
		200	1972.85	1580.74	1315.26

## Supplementary Materials

The following supporting information can be downloaded at: https://www.mdpi.com/article/10.3390/ma15010003/s1, Figure S1: Schematics of ZONRs-TFs growth using CBD, Figure S2: Cross-sectional FESEM images (right) and EDX spectra (left) of the undoped ZONRs-TFs, Figure S3: linear attenuation coefficient (µ) vs. the current of the tube for undoped ZONRs and doped with 1%, 2% and 3% of LA, the X-ray tube voltages 70 kVp, Figure S4: linear attenuation coefficient (µ) vs. the current of the tube for undoped ZONRs and doped with 1%, 2% and 3% of LN, the X-ray tube voltages 70 kVp. Figure S5: linear attenuation coefficient (µ) vs. the current of the tube for undoped ZONRs and doped with 1%, 2% and 3% of BN, the X-ray tube voltages 100 kVp. Table S1: The measured values of μ (cm^−1^) for Doped ZONRs-TF with wt 1%, 2% and 3% of LA the I = 100 mA, Table S2: The measured values of μ (cm^−1^) for Doped ZONRs-TF with wt 1%, 2% and 3% of LN the I = 100 mA, Table S3: The measured values of μ (cm^−^^1^) for Doped ZONRs-TF with wt 1%, 2% and 3% of BN the I = 100 mA.
