# Supplementary material for: Ionization Radiation Shielding Effectiveness of Lead Acetate, Lead Nitrate, and Bismuth Nitrate-Doped Zinc Oxide Nanorods Thin Films: A Comparative Evaluation
Source: Materials (Basel). 2021 Dec 21;15(1):3. doi: 10.3390/ma15010003 (PMC8746144; doi:10.3390/ma15010003)
Supplement: Supplementary file 1 [file materials-15-00003-s001.zip › materials-1425134-supplementary.pdf]

Ionization Radiation Shielding Effectiveness of Lead Acetate, Lead Nitrate, and Bismuth Nitrate-Doped Zinc Oxide Nanorods Thin Films: A Comparative Evaluation

[1] Mohamed. AS Al-Balushi[1], Naser M. Ahmed[1,*], Samer H. Zyoud[1,2,3], M.K.M. Ali[4], H. Akhdar[4,*], Osamah Aldaghri[4] and K.H. Ibnaouf[4]

[1] *School of Physics, Universiti Sains Malaysia, 11800 USM Penang, Malaysia.*

[2] *Department of Mathematics and Sciences, Ajman University, Ajman, United Arab Emirates.*

[3] *Nonlinear Dynamics Research Center (NDRC), Ajman University, Ajman, United Arab Emirates.*

[4] *Physics Department, College of Science, Imam Mohammad Ibn Saud Islamic University (IMSIU), P.O. Box 5701, Riyadh 11432, Saudi Arabia*

Corresponding author email: *nas_tiji@yahoo.com*

Supporting Information

Figure S1 shows the steps for preparing the Zinc oxide Nano-rods thin film (ZONRS-TF) on the glass substrate; First, prepare the Zink oxide (ZnO) 200 nm as a seed layer deposited on Glass substrates using reactive sputtering (RF). Second, prepare ZnO solution by using Zinc nitrate tetrahydrate ($Zn(NO_3)_2.4H_2O$) (ZNTH) and hexamethylene-tetraamine(HTMA), the solution was prepared by dissolving a 0.05 M equimolar ratio of zinc nitrate and (HMTA) together in 0.2 L of DI water and stirred for 1 hr at room temperature until getting a homogenous solution.

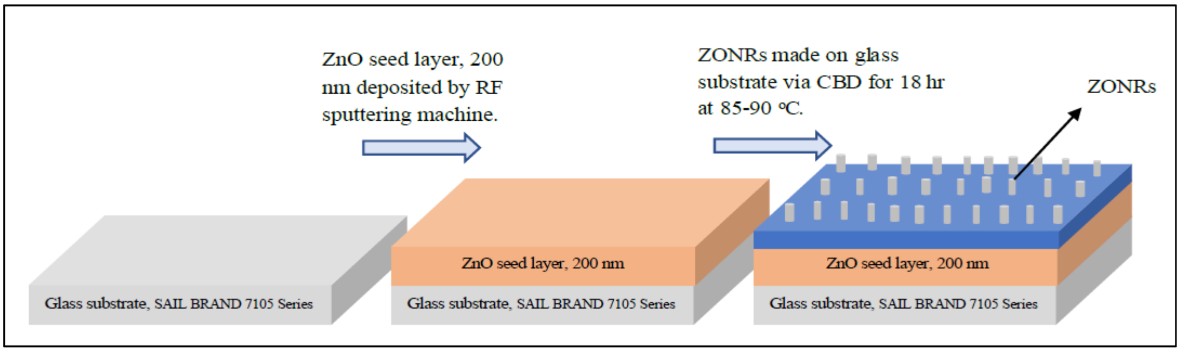

Figure S1. *Schematics of ZONRs-TFs growth using CBD.*

Figure S2 shows the Nano rods and thickness of the undoped ZnO NRs thin film deposited on the glass substrate by CBD for 18 hr at temperature (85-90 c°) and the molarity of ZnO is 0.05 mol/ml. The ZnO nanoparticles and thickness are distributed randomly on glass substrates, the cross-section was done by FESEM image to measure the thickness of ZONRs thin film, three values of thickness (4.080, 4.045 and 4.080 µm) were selected, and the thickness average of the three values was (4.068µm). The EDX measurement was used to determine the elements (zinc and oxygen), the amount of oxygen (O) was (44.79%) and for zinc (Zn) was (55.21%).

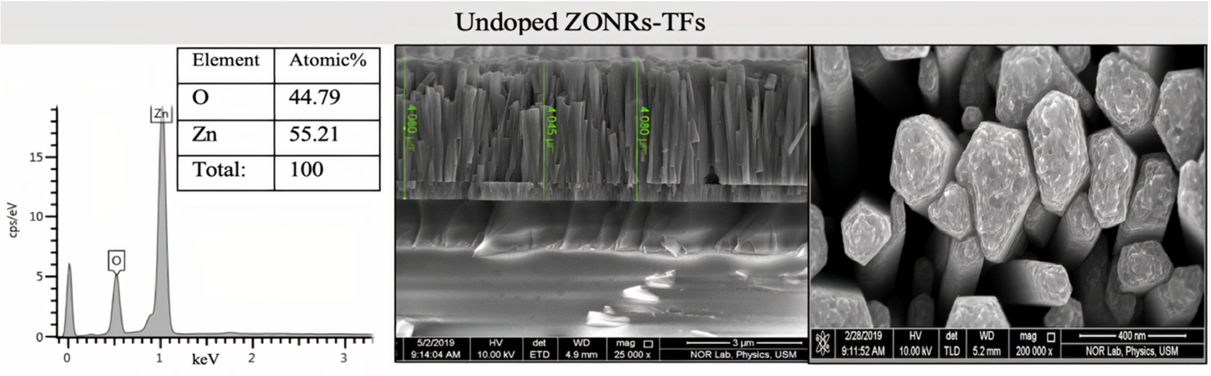

Figure S2. Cross-sectional FESEM images (right) and EDX spectra (left) of the undoped ZONRs-TFs.

This research was done using general diagnostic x-ray machine (brand: Toshiba X-ray KXO -50S) Was used, the initial x-ray beam intensity (I_o) was determined by directly measuring x-rays with the DIADOS diagnostic detector connected to semiconductor diagnostic dosimeter in the absence of the sample. Meanwhile the transmitted x-ray beam intensity (I) was taken with the sample placed on the detector. The distance between the x- rays' tube and the detector (SID) was set to 100 cm, the x-ray beams generated in the range of (50–100) kV tube voltage and the current range is (50, 100, 160 and 200 mA), it was chosen from the general diagnostic x-ray machine because this ranges is the normal ranges of the x-ray tube voltage used in general diagnostic imaging purposes.

The linear attenuation coefficient(μ) have been calculated as shown in table S1(1-3) by using the equation:

$$I = I_o \, e^{-\mu x}$$

$$\mu = \frac{(-\ln I/I_o)}{x}$$

Where:

I = Intensity of photon at thickness x,
I_0= Intensity of photon without absorber,
μ = coefficient of absorption and
x = thickness of absorber

Table S1. The measured values of μ (cm^{-1}) for Doped ZONRs-TF with wt 1%, 2% and 3% of LA the I=100 mA

a- Doped ZONRs-TF with wt 1%, 2% and 3% of LA the I=100mA, t= 0.25 s, coli-meter =10x10, SID=100cm,								
Sample	Doped 1% x= 242.8 nm		Doped 2% x= 10.385μm		Doped 3% x= 7.023μm		Undoped ZONRs x= 4.06μm	
KvP	I/I_o	μ (cm^{-1})	I/I_o	μ (cm^{-1})	I/I_o	μ (cm^{-1})	I/I_o	μ (cm^{-1})
50	0.714	13920.34	0.701	342.24	0.688	532.72	0.727	785.29
60	0.743	12275.17	0.726	308.48	0.717	473.90	0.752	702.02
70	0.759	11394.77	0.746	282.30	0.74	428.92	0.772	637.37
80	0.773	10639.51	0.763	260.59	0.753	404.12	0.788	586.84
90	0.785	10002.96	0.777	243.08	0.769	374.17	0.797	558.87
100	0.797	9376.06	0.791	225.87	0.784	346.65	0.804	537.33

Table S2. The measured values of μ (cm^{-1}) for Doped ZONRs-TF with wt 1%, 2% and 3% of LN the I=100mA

b- Doped ZONRs-TF with wt 1%, 2% and 3% of LN the I=100mA, t= 0.25 s, coli-meter =10x10, SID=100cm,								
Sample	Doped 1% x= 238.8 nm		Doped 2% x=6.01µm		Doped 3% x= 1.07µm		Undoped ZONRs x= 4.06µm	
KvP	I/I_o	µ (cm^{-1})	I/I_o	µ (cm^{-1})	I/I_o	µ (cm^{-1})	I/I_o	µ (cm^{-1})
50	0.71	14078.34	0.73	529.87	0.69	3490.78	0.727	785.29
60	0.74	12396.06	0.76	455.14	0.72	3096.80	0.752	702.02
70	0.76	11510.60	0.78	416.66	0.74	2807.67	0.772	637.37
80	0.77	10751.47	0.78	406.49	0.75	2642.69	0.788	586.84
90	0.79	9890.74	0.80	376.35	0.77	2452.00	0.797	558.87
100	0.80	9264.97	0.81	354.28	0.78	2271.33	0.804	537.33

Table S3. The measured values of µ (cm-1) for Doped ZONRs-TF with wt 1%, 2% and 3% of BN the I=100mA

c- Doped ZONRs-TF with wt 1%, 2% and 3% of BN the I=100mA, t= 0.25 s, coli-meter =10x10, SID=100cm,								
Sample	Doped 1% x=6.333 µm		Doped 2% x=3.318 µm		Doped 3% x=1.73µm		Undoped ZONRs x= 4.06µm	
KvP	I/I_o	µ (cm^{-1})	I/I_o	µ (cm^{-1})	I/I_o	µ (cm^{-1})	I/I_o	µ (cm^{-1})
50	0.73	503.09	0.73	962.10	0.71	2001.60	0.727	785.29
60	0.76	432.13	0.77	792.64	0.75	1662.90	0.752	702.02
70	0.78	382.80	0.79	707.80	0.75	1639.16	0.772	637.37
80	0.79	376.04	0.80	681.60	0.78	1420.07	0.788	586.84
90	0.81	341.36	0.81	637.68	0.79	1398.34	0.797	558.87
100	0.82	316.41	0.82	592.49	0.80	1289.85	0.804	537.33

S1.5. Effect of the Tube Current (mA) on the linear attenuation coefficient(μ)

Figures S3–S5 show that no effect of changing the current mA on the linear attenuation coefficient(μ) for ZONRs thin films. It illustrates that maintaining constant tube voltage (kVp) and exposure time and increasing tube current(mA) will increase the power applied to the filament, which heats up and releases more electrons to hit the target and the number of photons will increase without increasing their energy and the penetration ability of these photons through absorption materials and transmission intensity will be mostly constant.

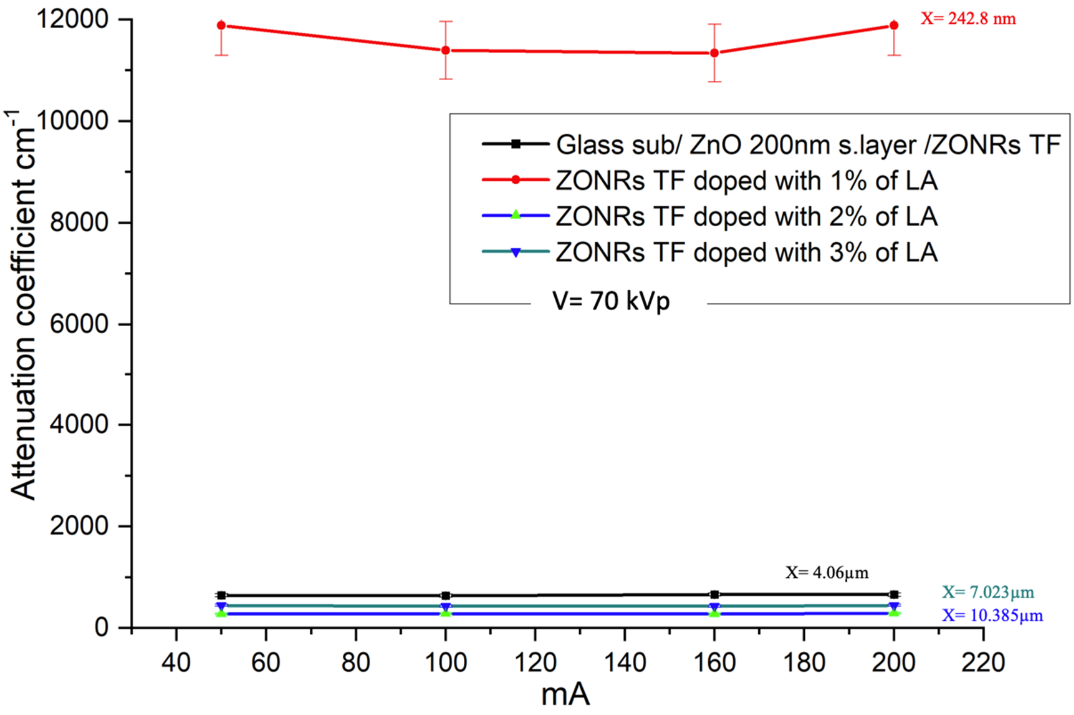

Figure S3. linear attenuation coefficient(μ) vs the current of the tube for undoped ZONRs and doped with 1%,2% and 3% of LA, the X-ray tube voltages 70 kVp

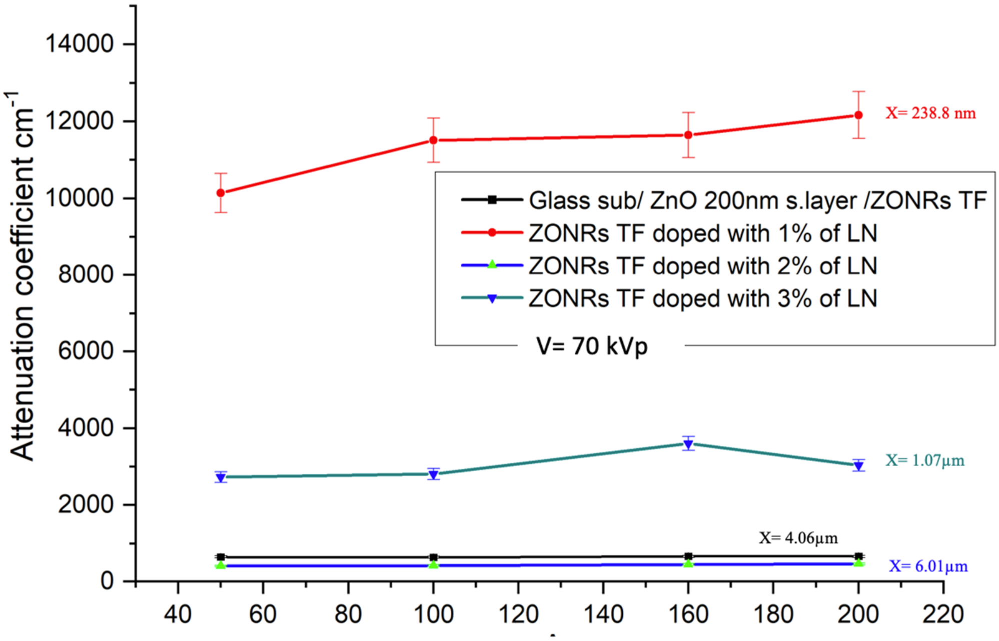

Figure S4. linear attenuation coefficient(μ) vs the current of the tube for undoped ZONRs and doped with 1%,2% and 3% of LN, the X-ray tube voltages 70 kVp

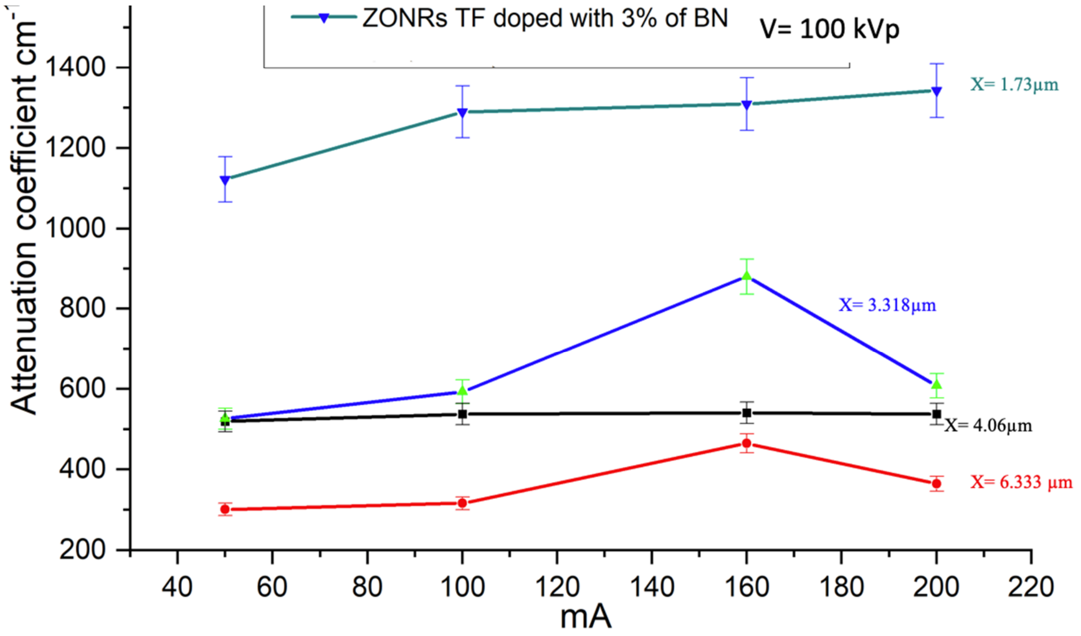

Figure S5. linear attenuation coefficient(μ) vs the current of the tube for undoped ZONRs and doped with 1%,2% and 3% of BN, the X-ray tube voltages 100 kVp
